# Dr. F. P. Abbot

**Published:** 1886-11

**Authors:** 


DR. F. P. ABBOT.

In the decease of Dr. Abbot, of Berlin, who died in Dresden, Oct. 11th, while on his way to Carlsbad, the dental profession of the world sustains a great loss. He was respected and beloved wherever American dentistry is known. During the greater part of his professional life he practiced abroad, and for many years occupied a prominent position in Berlin. By his age and honorable standing he had won the title of The Father of American Dentistry in Europe, and never was a name more worthily bestowed. Nor was it alone as a dentist that he was revered. He was a leader of the American Colony in Berlin, and his home was ever open to his compatriots who visited that city. For years he held regular receptions, at which all Americans who came properly vouched for were welcomed, and these entertainments were eagerly looked forward to by his countrymen in Berlin, for here might be met many people of distinction at home and abroad. Personally, he was one of the most genial and kind-hearted of men, and there are thousands who were his debtors for unnumbered courtesies. Professionally, he always occupied a prominent place, and when accomplished operators were few the fame of Dr. Abbot was widespread, and persons of every nationality might be found in his *salon*, visitors for professional treatment from all lands, attracted by the fame of his successful operations.

Never shall we forget the last time that it was our privilege to meet Dr. Abbot. He was at charming Schlangenbad, in Germany, where he had gone for needed rest and recuperation. We were at Wiesbaden, with his son-in-law, Dr. W. D. Miller, and together we drove across the country, among lovely vineyards and through a fairy valley, along the course of a winding stream. The whole route was a panorama of ever-changing loveliness, and the ride was brought to a fitting conclusion in the terraced amphitheatre of beautiful Schlangenbad.

Dr. Abbot greeted his visitors cheerily and heartily, although he was then suffering from a complication of disorders. The wonderful charm of the man was never more advantageously displayed than in the character of host, and as he that day entertained his friends at dinner he seemed the very embodiment of gentlemanly hospitality. His quiet, easy ways, his evident amiability and sweet, loving spirit, won every heart, and we wondered not that so many of his countrymen, when in trouble or distress in a strange land, should instinctively seek the counsel and assistance of Dr. Abbot. He has lived out his allotted space, and going hence he leaves behind him a name which exhales so sweet a perfume that its fragrance will long linger with those whom he has left behind.

